# *Torilis japonica* Extract Suppresses the Induction of Atopic Inflammation

**DOI:** 10.3390/ijms24032102

**Published:** 2023-01-20

**Authors:** Ji-Won Seo, Hyo-Jae Lee, Young-Mi Youk, Gun-He Nam, Young-Min Kim

**Affiliations:** Department of Biological Science and Biotechnology, College of Life Science and Nano Technology, Hannam University, Daejeon 34054, Republic of Korea

**Keywords:** atopic inflammation, *Torilis japonica*, HaCaT keratinocytes, 3D skin model, RhCE model

## Abstract

As one of the major intractable allergic disorders, atopic inflammation is commonly accompanied by itching, dry skin, and inflammation. Atopic inflammation deteriorates the quality of life and has no fundamental cure, so it is crucial to urgently explore and develop natural resources for long-term treatment without any side effects. This study aimed to verify *Torilis japonica* extract (TJE)’s relieving effect and mechanism against atopic inflammation using skin cells and skin equivalent models, as well as to investigate torilin’s effect (obtained from TJE) and other unknown components as marker compounds. Torilin concentration was verified in TJE using high-performance liquid chromatography and analyzed the unknown components using nuclear magnetic resonance spectroscopy. Furthermore, TJE’s cytotoxicity, regenerative effect, and cell cycle regulation effects were confirmed using skin cells with atopic inflammation (human dermal fibroblasts and HaCaT keratinocytes) by using TNF-α and IFN-γ treatments. Consequently, TJE was demonstrated to regulate TARC and CTACK expressions as chemokines and those of interleukin-4, -5, and -13 as cytokines related to atopic inflammation. TJE was further confirmed to affect the matrix metalloproteinase-1, -2, and -9 expressions, which are essential in skin damage. Lastly, this study confirmed TJE’s relieving effect against atopic inflammation through a 3D skin model and RhCE model using human dermal fibroblasts and HaCaT keratinocytes. These findings on atopic inflammation verified torilin’s relieving effects and TJE’s other components.

## 1. Introduction

Economic development has given rise to the generalized social phenomena of beauty care and rejuvenation. Owing to the increase in life expectancy, interest in preventing skin damage is increasing in the aging society, where both men and women equally spend considerable amounts of time and cost on skin care [1]. As a major intractable allergic disease, atopic inflammation is commonly accompanied by itching, dry skin, and inflammation [2,3,4]. Recently, skin barrier damage has emerged as atopic inflammation’s major cause. Skin barrier damage is easily caused by environmental factors, such as dry atmosphere and pollutants; life habits, such as excessive cleansing or bathing; and disorders, such as deteriorated lipid synthetic capacity of keratinocytes, resulting in atopic inflammation [5,6,7]. Atopic inflammation’s common clinical features are skin damage caused by severe itching, an increase in serum IgE levels, and eosinophil infiltration in inflamed areas [8,9]. Its major causes are cytokines secreted from Th1 and Th2 cells and IgE specific to T lymphocytes and antigens [10,11]. Particularly, high chemokine contents, such as CTACK and TARC, were identified in skin areas where atopic inflammation was induced. Therefore, these chemokines may play a core role in atopic inflammation [12,13,14]. Atopic inflammation induced by cytokines, such as TNF-α and IFN-γ, expresses CTACK and TARC as well as interleukins (ILs) in the affected skin cells [15,16,17,18,19,20]. Furthermore, matrix metalloproteinase (MMP) is a family of enzymes that intervenes in extracellular matrix and basement membrane [21,22] decomposition. They are classified into four subfamilies according to their function and structure: interstitial collagenase, stromelysin, gelatinase, and membrane-type MMP [23]. Numerous cells, including keratinocytes and fibroblasts, secrete MMPs. An increase in the skin’s MMP activity considerably damages intracutaneous collagen, resulting in collagen collapse, which is the key to skin damage [24]. Atopic inflammation affects MMP expression and induces long-term skin damage rather than simply inducing inflammation [25]. To relieve atopic inflammation symptoms that deteriorate the quality of life and have no fundamental treatment, the need to explore and develop natural resources for the use of long-term treatments with no side effects is warranted [26].

*Torilis japonica* (TJE) belongs to the family Umbelliferae. It is an edible plant known to be effective against conditions such as arthritis and skin diseases [27,28]. As a folk remedy, TJE has been used to treat skin diseases caused by fungi, viruses, and itching [29]. However, few studies have examined its effects on atopic inflammation and skin aging and damage.

This study aimed to verify the relieving effect provided by TJE and its mechanism against atopic inflammation using skin cells and a skin equivalent model. This study also further clarifies whether torilin, which is obtained from TJE, as well as its other unknown components, are marker compounds.

## 2. Results

### 2.1. Identifying Torilin in TJE

**Figure 1:**
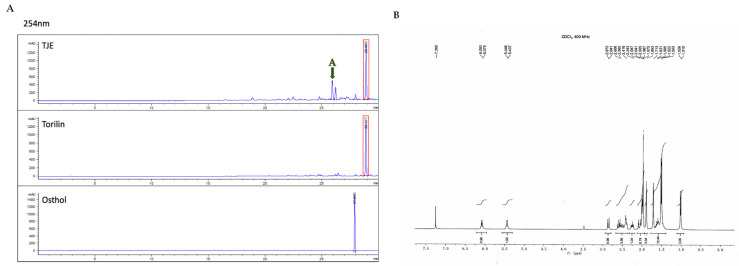
(**A**) HPLC profile of TJE, torilin, and osthol. Torilin was detected, but osthol was not detected in TJE (A: the unknown substance). (**B**) NMR profile of the unknown peak (substance) A.

Torilin and osthol (ChemFaces, Wuhan, China) were used as marker compounds while analyzing TJE using high-performance liquid chromatography (HPLC). Based on the torilin and osthol included in TJE (BioPapa), the contents of the new active materials were analyzed. For HPLC analysis, the torilin content was fixed (standard), while the osthol content was not. Furthermore, under similar analytical conditions, two new compounds were identified, which were separated and purified, based on the high peak (labeled as “A”) obtained (Figure 1A). Structural analysis was performed according to the analytical conditions used for NMR and based on the analyzed TJE’s HPLC peak. According to the analysis, A peak was identified as an effective component following separation and purification. Subsequently, NMR analysis (400 MHz) was performed to identify the new compound’s structure (Figure 1B). Experiments are currently ongoing for the clarification and verification of the new compound’s efficacy.

### 2.2. Verifying the Cytotoxicity of TJE

**Figure 2:**
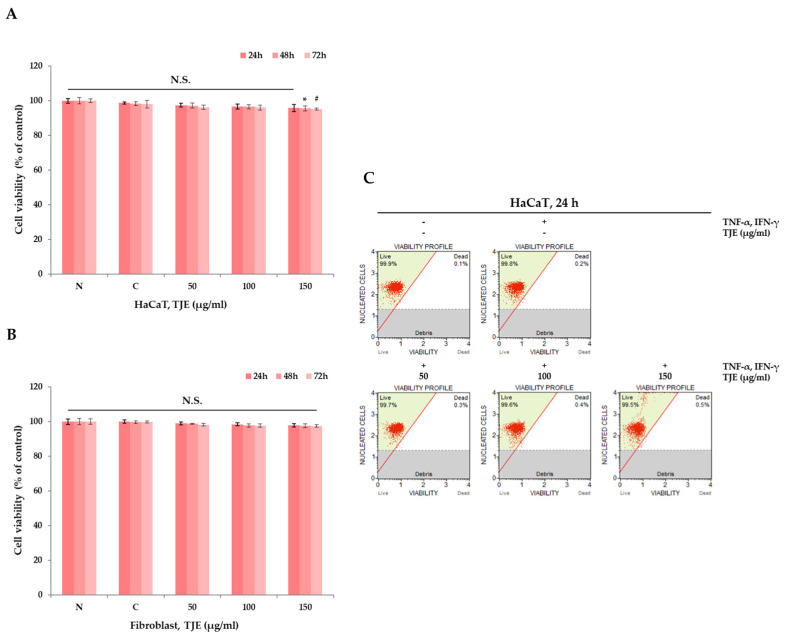
(**A**) Cell viability was measured by MTT assay. HaCaT keratinocytes were treated with variable concentrations of TJE (0–150 μg/mL) and certain cytokine (TNF-α (10 ng/mL) and IFN-γ (10 ng/mL)) for 24–72 h. (**B**) Cell viability was measured by MTT assay. Dermal fibroblasts were treated with variable concentrations of TJE (0–150 μg/mL) and certain cytokine (TNF-α (10 ng/mL) and IFN-γ (10 ng/mL)) for 24–72 h. (**C**) Cell viability assay using flow cytometry. The statistical analysis of the data was carried out by use of a *t*-test. *^,#^ *p* < 0.05 (each experiment, *n* = 3). N.S.; not significant. N: negative control, C: positive control (only TNF-α (10 ng/mL) and IFN-γ (10 ng/mL) treated group).

TJE cytotoxicity was examined using the MTT assay. By assessing MTT to formazan (purple pigment) conversion by living cells, changes in cell viability can be indirectly quantified according to test substance treatment. The cell strains used for testing were human dermal fibroblasts and HaCaT keratinocytes. Subsequently, cells were cultured under optimal testing conditions. The MTT test was conducted at least three times on each substance, and the mean of the results was used for interpretation. TJE cytotoxicity was quantified as a percentage according to its absorbance, depending on substance treatment, and by assuming the negative control group’s cell viability to be 100%. TJE cytotoxicity was confirmed using MTT assay after inducing atopic inflammation through TNF-α and IFN-γ. Compared to the negative control group, it was confirmed that there was no significance up to 150 μg/mL, and it was proven that there was no cytotoxicity (Figure 2A,B). Flow cytometry was used to validate this result. Even at a concentration of ≤150 μg/mL, cytotoxicity was nearly non-existent, which was similar to the effect observed in the MTT assay (Figure 2C). These results confirm TJE’s safety as it shows no cytotoxicity in the skin cells used.

### 2.3. Skin Cell Cycle and Regeneration Regulation by TJE

**Figure 3:**
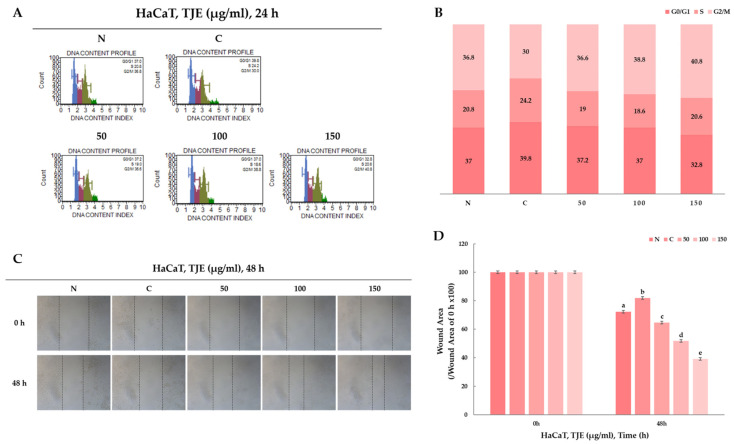
(**A**,**B**) TJE occurs in cell cycle progression. G2/M phase ratio increased compared with to the positive control. Cell cycle arrest effect was measured by flow cytometry. (**C**) Cell motility was measured using a wound-healing assay. Representative images of the cell migration were captured. (**D**) The graph of a wound-healing area using a wound-healing assay. The statistical analysis of the data was carried out by use of a *t*-test. ^b^ *p* < 0.01 and ^a,c,d,e^ *p* < 0.001 (each experiment, *n* = 3). N.S.; not significant. N: negative control, C: positive control (only TNF-α (10 ng/mL) and IFN-γ (10 ng/mL) treated group).

G2 cell cycle activity of human keratocytes was verified using the MUSE cell analyzer to demonstrate skin cell cycle regulation in relation to regeneration by TJE. Initially, cells were treated with TNF-α and IFN-γ to induce atopic inflammation, after which they were treated with TJE. As for the cell cycle activation effect, the change in G2 cycle activation was quantitatively analyzed according to the negative control group’s TJE treatment. The cell cycle activation verification method was used to examine the G2 cycle activation effect of TJE. It was confirmed that a certain level of the G2 cycle activity effect was exhibited according to TJE treatment when compared to the negative control group (Figure 3A,B). Furthermore, human keratinocytes (100% conflict) were uniformly wounded using a scratcher and were then treated with the test substance. Subsequent wound narrowing was qualitatively confirmed. All groups treated with TJE exhibited protection from atopic inflammation (Figure 3C,D). These findings confirm that TJE inhibits atopic inflammation to activate the cell cycle and positively affects skin cell regeneration.

### 2.4. TJE Treatment Regulated the Indicator of Atopic Inflammation in HaCaT Keratinocytes

**Figure 4:**
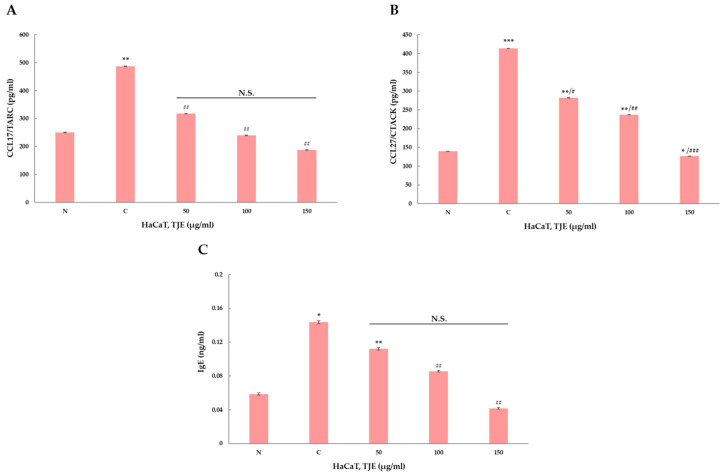
(**A**–**C**) Measurement of TARC, CTACK, and IgE through ELISA assay. TJE regulated TARC, CTACK, and IgE in HaCaT Keratinocytes. The statistical analysis of the data was carried out by use of a *t*-test. *^,#^ *p* < 0.05, **^,##^ *p* < 0.01 and ***^,###^ *p* < 0.001 (each experiment, *n* = 3). N.S.: not significant. N: negative control, C: positive control (only TNF-α (10 ng/mL) and IFN-γ (10 ng/mL) treated group).

**Figure 5:**
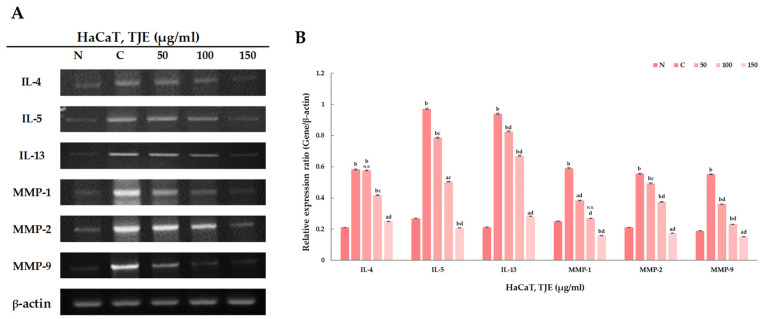
(**A**,**B**) The levels of IL-4, -5, -13 gene and MMP-1, -2, -9 gene were determined by RT-PCR. The β-actin probe served as protein-loading control. The statistical analysis of the data was carried out by use of a *t*-test. ^a^ *p* < 0.01 and ^b^ *p* < 0.001 vs. con (N). ^c^ *p* < 0.01 and ^d^ *p* < 0.001 vs. con (C) (each experiment, *n* = 3). N.S.: not significant. N: negative control, C: positive control (only TNF-α (10 ng/mL) and IFN-γ (10 ng/mL) treated group).

Atopic inflammation indicators’ degree of expression following TJE treatment was confirmed using enzyme-linked immunosorbent assay (ELISA). It was found that TARC, CTACK, and IgE’s degree of expression, which are atopic inflammatory indicators, decreased according to the increase in TJE treatment. As a result, TJE was found to increase the mitigating effect on atopic inflammation (Figure 4). Furthermore, changes in factor expression related to atopic inflammation according to TJE treatment were qualitatively analyzed via polymerize chain reaction (PCR) at the gene level. Atopic inflammation was artificially induced in HaCaT keratinocytes, and the changes in the expressions of the factors related to atopic inflammation were verified with TJE treatment. Based on IL-4, -5, and -13 level assessments, a high TJE concentration produced a more relieving effect against atopic inflammation. Furthermore, MMP-1, -2, and -9 expressions decreased in a concentration-dependent manner, which suggests the excellent skin regenerative and relieving effects of TJE against atopic inflammation (Figure 5).

### 2.5. TJE Was Associated with Anti-Atopic Dermatitis Effect in an Ex Vivo Model

**Figure 6:**
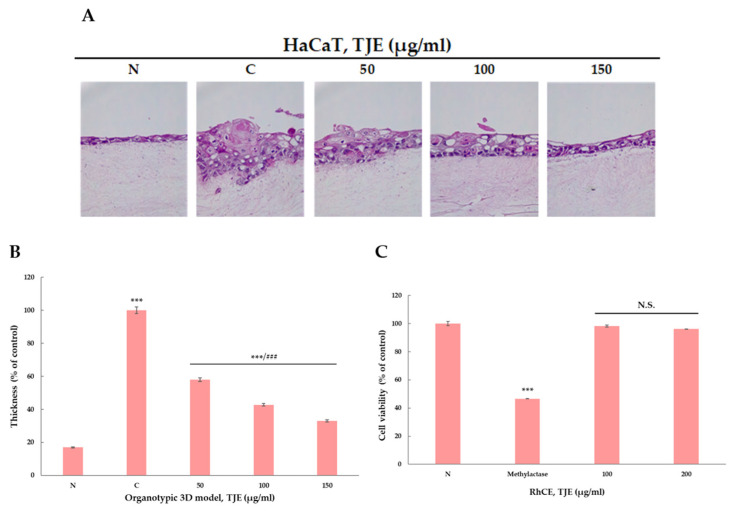
(**A**) The organotypic three-dimensional models using HaCaT keratinocytes and dermal fibroblasts were stained for H&E as red and pink areas and represented by a microscope (Carl Zeiss, USA). The photographs were taken at a magnification of ×200. (**B**) Measurement of the keratin layer. The layer was counted from randomly selected fields of each section using the ImageJ program. (**C**) In vitro RhCE assay (eye irritation test) was tested for TJE (100 μg/mL and 200 μg/mL) using the OCL 200 EIT. Methylactase was used as positive control. The statistical analysis of the data was carried out by use of a *t*-test. ***^,###^ *p* < 0.001 (each experiment, *n* = 3). N.S.: not significant. N: negative control, C: positive control (only TNF-α (10 ng/mL) and IFN-γ (10 ng/mL) treated group).

One of the common features of patients with atopic inflammation is excessive skin surface keratinization, which results in the failure of timely treatment against inflammation in the inner skin layer. In this study, TJE was found to relieve inner skin layer inflammation by inducing the normal skin keratin turnover maintenance and normalizing the skin surface damaged due to atopic inflammation. The organotypic cell culture test was conducted at least thrice, and the mean of the resulting values was used for interpretation. PBS was used in the negative control, and atopic inflammation was induced in the positive control. The induced cell culture was treated with TNF-α and IFN-γ at adequate concentrations. To quantify the TJE effect, changes in the keratin layer’s thickness of the TJE treatment group were compared with those of the positive control group. As a result, the positive control group where atopic inflammation was induced had an excessive keratin layer, resulting in atopic skin inflammation. TJE treatment inhibited excessive keratin secretion to maintain keratin layer thickness in the negative control group (Figure 6A,B). Furthermore, the reconstructed human cornea-like epithelium (RhCE) model was used for verifying the safety of the TJE 3D culture model. The model was designed to cultivate human corneal cells to have layered structures similar to human corneas and highly differentiated epithelial cells. This model comprised at least three layers of living cells and a non-keratinized surface to form a corneal structure similar to that observed in a living body. The RhCE model test was conducted at least thrice, and the mean of the results was used for interpretation. PBS was used as the negative control, and methylacetate was utilized as the positive control. Each test substance’s safety was quantified (percentage) according to the difference in absorbance following treatment with the test substance, assuming a cell viability of 100%. As a result, the TJE group’s cell viability showed no significant difference compared with that of the negative control group (Figure 6C). These findings suggested that the TJE exhibited no toxicity in the actual skin layer and instead relieves atopic inflammation.

## 3. Discussion

There have been various attempts to treat patients based on their clinical conditions as the mechanism underlying atopic dermatitis is complex and its phenotypes are highly diverse [30]. Some of these attempts have continuously used products containing medically verified components, which are referred to as cosmeceuticals (cosmetic and pharmaceutical). Cosmeceuticals are future-oriented skin improvement products that can be procured without prescription and have a wider application range and usage than functional cosmetics [31]. In line with the naturalism and environmental-friendliness trends, the effective ingredients of cosmeceuticals, based on the utility of naturally derived components, as well as chemical ones, have extended to various natural ingredients, including herbal medicines, leading to the golden age of natural and oriental medicine [32].

In line with these trends, effectively using treatments based on useful naturally derived therapeutic agents specifically tailored for patients with atopic dermatitis will be possible. Currently, IgE is the most promising atopic dermatitis biomarker. Based on the IgE results, the atopic dermatitis expression has been classified as either intrinsic or exogenic. However, patients with severe atopic dermatitis showed an increase in atopic inflammation activation-regulated chemokine (e.g., TARC and CATACK) in addition to IgE. Furthermore, IL and MMP inhibition, which have been reported to intervene in allergic inflammation and skin fibrosis in atopic dermatitis, act as an essential factor for relieving atopic dermatitis [33,34].

This study has been conducted to acquire effective and active materials from TJE, a natural ingredient, to relieve the symptoms of atopic dermatitis. The present study’s results can prove extremely advantageous considering the current increased need for naturally derived materials. Moreover, torilin was used as the marker compound, which has been found to have effects by many studies, thus verifying a higher torilin content in TJE than other natural materials and its subsequent effects, which distinguishes torilin from other extracts and materials. TJE, with its relieving effect on atopic dermatitis, as demonstrated through this study, further eliminates the fundamental cause of symptoms and potentially recovers healthy and smooth skin beyond merely relieving superficial symptoms. Furthermore, this study’s results have potentially wide applications to other skin disease models that share the same disease cause, showing possible extendibility to quasi-drug development for improving skin inflammation and treating atopic dermatitis symptoms, as well as to cosmetic material development.

## 4. Materials and Methods

### 4.1. Plant Material and Preparation of TJE

Dried whole fruit of *Torilis japonica* was purchased from Na-num Pharmacy (Kyung-buk, Republic of Korea). It was grown and dried in Kyung-buk, Republic of Korea. It was ground using a blender and was extracted with purified water at 120 °C and 1.5 AP (atmospheric pressure) for 2 h. Then, the extraction of *Torilis japonica* powder was optimized via a freeze-drying method and stored at −C80 °C.

### 4.2. High-Performance Liquid Chromatography (HPLC) and NMR Analysis

#### 4.2.1. *Torilis japonica* Extract HPLC Analysis

*Torilis japonica* extract (TJE) was dissolved in purified water and active substances were investigated using HPLC. The analysis conditions are as follows (Table 1).

#### 4.2.2. The Marker Components HPLC Analysis

The marker components of *Torilis japonica* (Torilin and Osthol) were investigated using HPLC. The analysis conditions are as follows (Table 2).

#### 4.2.3. NMR Analysis

To analyze the unknown substance (peak A), NMR analysis was performed using a Bruker Avance II+-500 FT-NMR Spectrometer (Bruker, Billerica, MA, USA). The analysis conditions are as follows (Table 3).

### 4.3. Cell Culture

HaCaT keratinocytes and human dermal fibroblasts were obtained from the American Type Culture Collection (ATCC; Rockville, MD, USA). The cells were grown in DMEM medium (HyClone, Laboratories Inc., Logan, UT, USA) containing 10% Fetal bovine serum (HyClone, Laboratories Inc., Logan, UT, USA) and 1% antibiotics (100 mg/streptomycin, 100 U/mL penicillin) at 37 °C in a 5% CO_2_ incubator.

### 4.4. Cell Proliferation Assay (MTT Assay)

Cells were seeded at 1.9 × 10^5^ cells/mL in a 24-well plate for 24 h and were incubated with TJE (50–150 μg/mL) for 24 h. Certain cytokine (TNF-α (10 ng/mL) and IFN-γ (10 ng/mL)) were pre-treated for 1 h. Following incubation with the TJE, the cells were incubated with MTT solution (5 mg/mL) for 120 min. Then, 150 μL of DMSO was added to dissolve the purple formazan crystals. The optical densities of the solutions were quantified at a 595 nm wavelength by using a FLUOstar Omega (BMG labtech, Ortenberg, Germany).

### 4.5. Cell Migration Assay

Cells were seeded in a 6-well plate and cultured until confluent. Then, by using a 200-microliter pipette tip (Corning, NY, USA) a straight scratch was made, simulating a wound area, and was incubated with TJE (50–150 μg/mL) for 48 h. The wound healing area was photographed under a microscope (Carl Zeiss, Oberkochen, Germany). The photographs were taken at a magnification of ×200.

### 4.6. Cell Viability Assay (Flow Cytometry)

Cells were seeded at 9.5 × 10^5^ cells/mL in a 6-well plate for 24 h. The cells were treated with TJE (50–150 μg/mL), certain cytokine (TNF-α (10 ng/mL), and IFN-γ (10 ng/mL)) for 24 h. Following incubation, the cells were re-suspended with PBS, and the reagent (consisting of DNA-binding dye and membrane-permeant, DNA-staining dye) was added. This parameter discriminated the live cells and dead cells were acquired using a Muse™ Cell Analyzer (MilliporeSigma, Burlington, MA, USA), and the results were read.

### 4.7. Determination of Cell Cycle (Flow Cytometry)

Cells were seeded at 9.5 × 10^5^ cells/mL in a 6-well plate for 24 h. The cells were treated with TJE (50–150 μg/mL) and certain cytokines (TNF-α (10 ng/mL) and IFN-γ (10 ng/mL)) for 24 h. Following incubation, the cells were re-suspended with PBS, and 0.2 mL of cold-70% ethanol was slowly added. After an incubation period of at least 3 h, at −20 °C, the fixed cells were analyzed in a Muse™ Cell Analyzer (MilliporeSigma, Burlington, MA, USA). The fixed cells were mixed with 200 μL of premixed reagent (consisting of the nuclear DNA intercalating stain PI (propidium iodide) and RNAse) and incubated for 30 min at room temperature in the dark. Then, the stained cells were analyzed in a Muse™ Cell Analyzer (MilliporeSigma, Burlington, MA, USA). Based on the differential DNA content, the PI discriminates cells at different stages of the cell cycle.

### 4.8. ELISA Assay

Cells were seeded at 9.5 × 10^5^ cells/mL in a 6-well plate for 24 h. The cells were treated with TJE (50–150 μg/mL) and certain cytokine (TNF-α (10 ng/mL) and IFN-γ (10 ng/mL)) for 24 h. Following incubation, cell culture media were centrifuged at 2000× *g* for 10 min to remove debris. The supernatants were collected and added to an immobilization antibody-coated 96-well plate. Then, the antibody cocktail was added and incubated at room temperature. The well was aspirated and washed, and TMB substrate was added; the incubation time was 10 min. A stop solution was added and the results were read using a FLUOstar Omega (BMG labtech, Ortenberg, Germany).

### 4.9. Reverse Transcription Polymerase Chain Reaction (RT-PCR)

Total RNA was extracted using RiboEx according to the manufacturer’s instructions, and cDNA was generated using a ReverseAids cDNA synthesis kit (Bioneer, Daejean, Republic of Korea) according to the manufacturer’s instructions. RT-PCR was performed with the following temperature profile: pre-denaturation at 95 °C for 10 min, followed by 35 cycles of denaturing at 95 °C for 30 s, annealing at 60 °C for 30 s, extension at 72 °C for 30 s, and a final extension step at 72 °C for 10 min. The specific primer sequence for amplification was as followed (Table 4).

### 4.10. RhCE Assay (Eye Irritation Test)

The ocular irritation potential of TJE was examined using the EpiOcular Eye Irritation Test (MatTek Corp., Ashland, MA, USA). Following incubation in 5% CO_2_ at 37 °C for 24 h, the RhCE assay was pre-wet with 20 μL of D-PBS (Sigma Aldrich, St. Louis, MO, USA) for 1 h. Then, 50 μL of FBG was applied to the pre-wet RhCE assay and incubated in 5% CO_2_ at 37 °C for 1 h. Tissues in the RhCE assay were washed with D-PBS, soaked with completed media, and incubated in 5% CO_2_ at 37 °C for 1 h. To investigate cytotoxicity, the RhCE assay was incubated with MTT solution (5 g/mL) for 3 h. After incubation, the absorbance of formazan was measured at 595 nm on a FLUOstar Omega (BMG Labtech, Ortenberg, Germany).

### 4.11. Organotypic 3D Cell Culture Model

The 6-well plates were pre-coated with type I collagen and incubated for 45 min at 37 °C in a 5% CO_2_ incubator. The 1 × 10^6^ cells/mL human dermal fibroblasts were seeded on a size cell culture insert plate (0.3-μm pore) and incubated with Matrigel and type I collagen mixture for 24 h. Afterward, the cell mixture was detached from the insert plate and incubated for 3 days in the complete medium. Then, the matrigel formation medium was placed in the bottom well and cultured for 2 weeks while the medium was changed with TJE (50–150 μg/mL) and certain cytokine (TNF-α (10 ng/mL) and IFN-γ (10 ng/mL)) every 2 days.

### 4.12. H&E (Harris’ Hematoxylin and Eosin) Stain

The fixed organotypic 3D cell cultures sections were de-paraffinized and re-hydrated in xylene, absolute alcohol, then washed with distilled water and stained with Harris hematoxylin solution and eosin–phloxine solution. The nuclei should be blue and the cytoplasm (keratin layer) pink to red. The sections were photographed under a microscope (Carl Zeiss, Oberkochen, Germany). The photographs were taken at a magnification of ×200.

### 4.13. Statistical Analysis

All the experiments were repeated at least three times and analyzed using *t*-tests (SPSS 20.0; IBM SPSS, Armonk, NY, USA). *p* < 0.05 was considered to indicate a statistically significant difference.

## Figures and Tables

**Table 1:** *Torilis japonica* extract HPLC Analysis Conditions.

HPLC Condition	
HPLC	Agilent 1200 series
Column	YMC J’sphere ODS-H80 (4.6 × 250 mm), S-4 μm, 8 nm
Detector	UV (254 nm)
Flow	1 mL/min
Mobile phase(A: 0.05% TFA in Water, B: MeOH)	80:20 (0-30.0 min), 0:100 (30.0 min)

**Table 2:** The marker components HPLC Analysis Conditions.

HPLC Condition	
HPLC	Agilent 1200 series
Column	InertSustain C18 column (5 μm, 4.6 × 150 mm)
Detector	UV (254 nm)
Flow	1 mL/min
Mobile phase	20% to 100% MeOH in Water over 8.0 min followed by 100% MeOH to 13.0 min

**Table 3:** NMR Analysis Conditions.

NMR Condition
Identification by ^1^H-NMR to Consistent with the above structure
Purity tested by HPLC, ^1^H-NMR (95%)

**Table 4:** Specific primer sequence.

Gene	Primer Sequence
IL-4	F: 5′-GCCACCATGAGAAGGACACT-3′
	R: 5′-ACTCTGGTTGGCTTCCTTCA-3′
IL-5	F: 5′-GAGACCTTGGCACTGCTTTC-3′
	R: 5′CAGTACCCCCTTGCACAGTT-3′
IL-13	F: 5′-GTACTGTGCAGCCCTGGAAT-3′
	R: 5′-TTTACAAACTGGGCCACCTC-3′
MMP-1	F: 5′-GGTCTCTGAGGGTCAAGCAG-3′
	R: 5′-AGTTCATGAGCTGCAACACG-3′
MMP-2	F: 5′-ATGACAGCTGCACCACTGAG-3′
	R: 5′-ATTTGTTGCCCAGGAAAGTG-3′
MMP-9	F: 5′-TTGACAGCGACAAGAAGTGG-3′
	R: 5′-GCCATTCACGTCGTCCTTAT-3′

## Data Availability

Not applicable.
